# A Literature Review of the Therapeutic Perspectives of Sodium-Glucose Cotransporter-2 (SGLT2) Inhibitor-Induced Euglycemic Diabetic Ketoacidosis

**DOI:** 10.7759/cureus.29652

**Published:** 2022-09-27

**Authors:** Khyati Patel, Arun Nair

**Affiliations:** 1 Internal Medicine, Surat Municipal Institute of Medical Education and Research, Surat, IND; 2 Pediatrics, Saint Peter’s University Hospital, Somerset, USA

**Keywords:** precipitating factors for euglycemic dka, withholding period of sglt2 inhibitor, sodium-glucose cotransporter 2 (sglt-2) inhibitors, dka with normal blood glucose, euglycemic dka, treatment of euglycaemic diabetic ketoacidosis, diabetes mellitus type 2, sglt 2 inhibitor, diabetic ketoacidosis (dka), euglycaemic diabetic ketoacidosis

## Abstract

Euglycemic diabetic ketoacidosis (DKA), a side effect of sodium-glucose cotransporter-2 (SGLT2) inhibitors, is a triad of high metabolic anion gap acidosis, raised serum and urine ketones, and serum glucose <250 mg/dl. SGLT2 inhibitors cause a carbohydrate deficit by glucosuria, which leads to an increased glucagon/insulin ratio, the metabolic shift from glucose to lipid utilization causing ketogenesis, and hence euglycemic DKA. Additional factors like the ketogenic diet, illness, surgery, and pregnancy contribute to precipitating these episodes.

Keywords search included “Euglycemic DKA and SGLT2 inhibitors” in PubMed and Google Scholar, to compile data from existing articles that provide information on the withholding and restarting period of the drug after a euglycemic DKA episode.

SGLT2 inhibitors, used in the treatment of type 2 DM, have an average half-life of 11-13 hours, so most articles suggested withholding the drug three days before any elective surgery but some articles suggested a longer withholding period based on other precipitating factors contributing to euglycemic DKA. Hence, we came up with patient inclusion criteria and concomitant therapies review that we need to consider in making this decision. In addition, a multidisciplinary approach is required when laying out guidelines for restarting the drug to have a unanimous approach.

After reviewing the existing literature, it is clear that concrete guidelines are required to decide on drug withholding and restarting periods after a euglycemic DKA episode, as they vary among different institutions and different specialties. We believe it is crucial to consider patient inclusion criteria and concomitant therapies in forming those guidelines.

## Introduction and background

Euglycemic diabetic ketoacidosis is defined as a triad comprising high anion gap metabolic acidosis with positive serum and urine ketones when serum glycemic levels are <250 mg/dL. It is one of the side effects of sodium-glucose cotransporter-2 (SGLT2) inhibitors. In 2015, the FDA released drug safety warnings about the risk of euglycemic DKA with the use of SGLT2 inhibitors [1]. SGLT2 inhibitors, also known as gliflozins, first marketed in 2013, are a class of medications used to treat type 2 diabetes mellitus. Sodium-glucose cotransporter (SGLT) is a transmembrane protein and, as its name suggests, cotransports sodium and glucose across the cell membrane. Although there are six isoforms, two important ones are type 1 and 2, with the former located one intestinal cells, and the latter mostly on the apical cells of the proximal convoluted tubules in the kidney. These transporters are driven by the electrochemical gradient generated by the basolateral sodium/potassium-ATPase pump, and SGLT2 aids in the reabsorption of glucose from the proximal convoluted tubule (PCT). SGLT2 inhibitors work by inhibiting SGLT2 in the PCT, to prevent the reabsorption of glucose and facilitate its excretion in urine.

**Figure 1:**
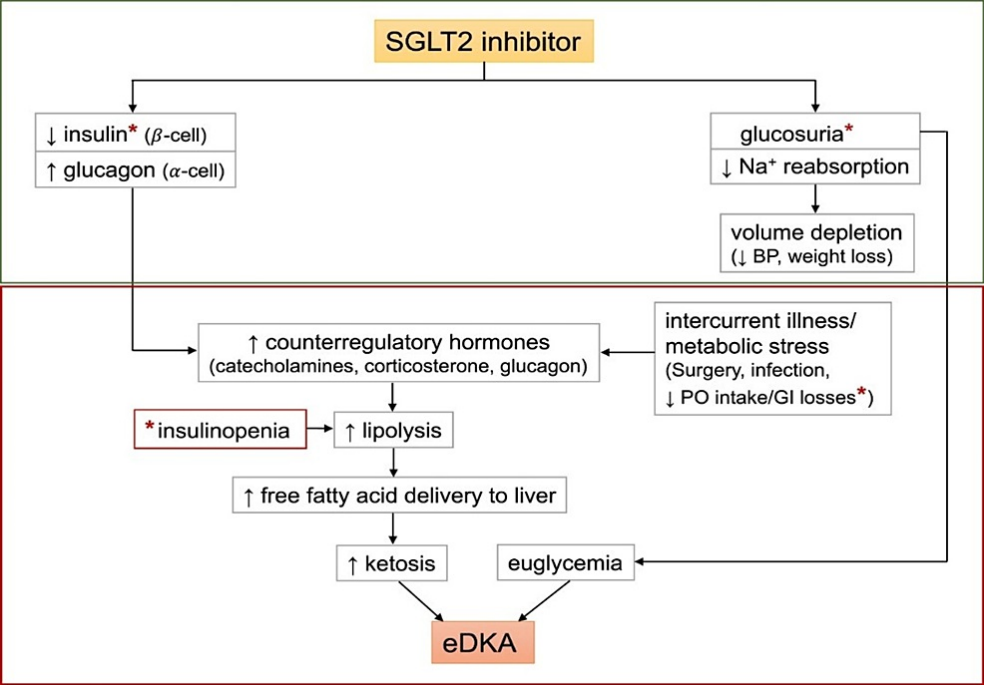
Proposed role of sodium-glucose cotransporter 2 (SGLT2) inhibition in euglycemic diabetic ketoacidosis (eDKA). Classic DKA results from insulin deficiency (absolute or relative) and concurrent increase in counter-regulatory hormones leading to ketosis, hyperglycemia, and osmotic diuresis. In contrast, SGLT2 inhibitor therapy in a well-compensated individual at baseline causes glucosuria, mild volume depletion, and lower serum glucose levels, associated with decreased insulin secretion (green box). During times of intercurrent illness and/or metabolic stress (e.g., surgery or gastrointestinal illness), decreased carbohydrate intake coupled with lower serum glucose levels can further depress insulin secretion. This can ultimately lead to eDKA (red box). © <2020>. This manuscript version is made available under the CC-BY-NC-ND 4.0 license https://creativecommons.org/licenses/by-nc-nd/4.0 *Possible pathways of carbohydrate deficiency and causes of insulinopenia. Wang et al. explained this figure [2]. eDKA - euglycemic diabetic ketoacidosis; DKA - diabetic ketoacidosis; SGLT2 - sodium-glucose cotransporter 2; BP - blood pressure; PO - per oral

The proposed mechanism of SGLT2 inhibitor-associated euglycemic diabetic ketoacidosis implicates glucosuria leading to decreased plasma glucose levels and decreased insulin release. A carbohydrate deficit, decreased insulin levels, and increased glucagon release leads to the upregulation of lipolysis and ketogenesis. Decreased carbohydrate intake and/or deficit related to glucosuria leads to euglycemia in these patients. Wang et al. illustrated this mechanism in Figure 1 [2].

Latent autoimmune diabetes of adults (LADA) has been described by WHO as ‘slow evolving immune-related diabetes.’ The Immunology for Diabetes Society (IDS) has specified three criteria for the diagnosis of LADA: 1. Age greater than 35 years; 2. Positive autoantibodies to islet beta cells; 3. Insulin independence for at least the initial six months after initial diagnosis.

LADA results in insulin deficiency, which is exaggerated in patients taking SGLT2 inhibitors, making them more prone to euglycemic DKA.

Erondu et al. mentioned that LADA may be misdiagnosed as type 2 DM, suggesting it is important to screen the patients for LADA before prescribing them with SGLT2 inhibitors [3]. Finucane et al. suggested there could be a rare variant of the SGLT family that has more than a normal affinity for SGLT2 inhibitors, which could account for SGLT2 inhibitor-induced euglycemic DKA [4]. Conditions like anorexia, gastroparesis, fasting, use of a ketogenic diet, and alcohol use disorder can lead to states of carbohydrate starvation and resultant ketosis. Additional triggers for eDKA include pregnancy, pancreatitis, glycogen storage disorders, surgery, infection, cocaine toxicity, cirrhosis, and insulin pump use. T1DM who underwent bariatric surgery patients experience DKA in over 20% of postoperative cases and may be especially prone to eDKA [5].

This study aims to guide physicians regarding the therapeutic perspectives of SGLT2 inhibitor-induced euglycemic DKA through the following objectives: 1. It tries to answer the dilemma of whether to continue the drug or not when patients experience its side effect- euglycemic DKA while also giving information about the drug withholding time based on existing literature; 2. It also cautions about drug-drug interactions which can precipitate euglycemic DKA, hence the review of concomitant therapies is also an important factor to consider.

Materials and methods

A search was done using PubMed and Google Scholar with the following words: “Euglycemic DKA and SGLT2 inhibitors”, “SGLT2 inhibitors induced Euglycemic DKA”, “Management of Euglycemic DKA”, and “Causes of Euglycemic DKA”. We have included case reports, case series, literature reviews, and systematic reviews from 2009 to 2022, those in English, those involving guidelines on drug withholding as well as the drug restarting period after an episode of euglycemic DKA. These articles were reviewed and those that fit the inclusion criteria of keywords and study aims aforementioned were extracted and compiled.

Pathophysiology of Euglycemic DKA

Euglycemic DKA is caused by SGLT2 inhibitors via various factors ultimately leading to an increased glucagon/insulin ratio. Normally, glucosuria occurs when blood glucose concentration crosses the threshold of 225 mg/dl. SGLT2 inhibitors act on proximal convoluted tubules of the kidney, block glucose reabsorption even at physiologic serum glucose concentrations, and cause glucosuria and osmotic diuresis even in normoglycemia [6]. This results in a carbohydrate deficit, which causes increased glucagon levels. It also directly stimulates pancreatic cells to increase glucagon release as well as increases ketone reabsorption. Increased glucagon/insulin ratio results in lipolysis and increased free fatty acid levels, which undergo beta-oxidation and hepatic ketogenesis and get converted into ketone bodies like acetone and 3-hydroxybutyrate. Stress, illness, surgery, fasting, and ketogenic diets result in increased levels of counterregulatory hormones like cortisol and catecholamines, which ultimately causes an increased glucagon/insulin ratio [5,7,8]. Pregnancy is a physiologic state of hypoinsulinemia and the human lactogen hormone produced during pregnancy causes insulin resistance, vomiting, and starvation resulting in ketogenesis, in addition to that respiratory alkalosis, which is also common in pregnancy due to stimulation of the respiratory center by progesterone, which results in bicarbonate excretion and acidosis develops [5]. It is important to note that the fetus and placenta use large amounts of maternal glucose as a major source of energy, and this leads to decreased maternal fasting glucose [9]. Hence, pregnancy is an important precipitating factor for euglycemic DKA. Alcohol consumption can result in pancreatic destruction, which in turn leads to decreased gluconeogenesis and decreased glycogen stores. In addition to that, vomiting and carbohydrate starvation result in accelerated lipolysis, ketoacidosis, and euglycemic DKA [5].

Pharmacokinetics of SGLT2 Inhibitors

SGLT2 inhibitors share similar pharmacokinetic characteristics with rapid oral absorption, extensive hepatic metabolism via glucuronidation, and renal as well as fecal elimination. Almost all of them have long half-lives hence once daily administration is adequate [10]. Hence, these drugs are contraindicated in case of renal failure and caution must be taken in patients having hepatic dysfunction. The area under the curve and Cmax increase proportionately in a dose-dependent manner. Drugs are distributed largely to the kidney and are found in smaller amounts in the liver, stomach, and small and large intestines. The lipophilicity of the drugs is different, it increases from empagliflozin< dapagliflozin< canagliflozin. Individual drug pharmacokinetics are shown in Table 1 [11-14].

**Table 1:** Individual SGLT2 inhibitor pharmacokinetics SGLT2 - sodium-glucose cotransporter-2; eGFR - estimated glomerular filtration rate

Drug	Half-life (t1/2)	Time to peak action	Plasma protein binding	Bioavailability	Contra-indication
Canagliflozin	11-13 hr	1-2 hr	99%	~65%	eGFR<30 ml/min/1.73m2
Dapagliflozin	~13 hr	1-1.5 hr	78%	~78%	eGFR <60 ml/min/1.73m2
Empagliflozin	~13 hr	1.5 hr	86%	~75%	eGFR <45 ml/min/1.73m2
Ertugliflozin	11-17 hr	0.5-1.5 hr	-	70-90%	eGFR<30 ml/min/1.73m2
Ipragliflozin	15-16 hr	1.5 hr	-	~90%	(Only approved in Japan)
Tofogliflozin	5-6 hr	0.5-1.5 hr	-	~97.5%	(Only approved in Japan)

Current Guidelines for the Management of Euglycemic DKA

Initially, the diagnosis of euglycemic DKA should be made based on positive serum and urine ketone levels along with pH < 7.3 and raised anion gap with serum glucose < 250 mg/dl. Management includes stopping SGLT2 inhibitors immediately, intravenous fluid resuscitation, continuous intravenous insulin infusion, 5% dextrose infusion, and potassium monitoring [5]. Initially, we use isotonic saline (0.9% NaCl) for fluid resuscitation for one to two hours at a rate of 1-1.5 L/hr according to the American Diabetes Association. Continuous IV insulin infusion should be started along with fluids at the rate of 0.05-0.1 U/kg/hr and to avoid hypoglycemia and hasten the clearance of ketone bodies, we should start the patient on 5% dextrose solution as well. After euglycemic DKA resolves, we can transfer the patient from IV insulin to SC insulin by keeping an overlap of two hours between them [15]. While the patient is on insulin infusion, it is important to monitor potassium levels as there is a total body potassium deficit and insulin tends to drive potassium into the cell. So, if potassium levels go below 3.3 mg/dl, we should stop insulin and initiate potassium replacement. Close monitoring of the patient’s blood glucose, electrolyte, and anion gap levels should be done every two to four hours and fluid resuscitation should be continued until the anion gap closes and acidosis resolves. The literature recommends not using bicarbonates in the treatment [5].

## Review

Discussion

SGLT2 inhibitors like canagliflozin (Invokana), dapagliflozin (Farxiga), and empagliflozin (Jardiance) have been approved by the FDA for use in type 2 diabetes mellitus. The average half-lives of these drugs range from 11-13 hours. According to the pharmacokinetics of the drug, it takes five half-lives for 97% of the drug to be eliminated from the body. Hence, the current recommendation by FDA, which was revised in 2022, is to stop these drugs three days before surgery [7]. Similarly, an International Consensus Review on SGLT2 inhibitors suggested stopping the drug three days before surgery [16]. Lau et al. also supported this idea by mentioning in their article that in major elective surgical procedures, SGLT2 inhibitors should be discontinued three days preoperatively, with the last dose taken no less than 55-65 hours before the surgery [17].

However, it was suggested by Pace et al. to stop the drug for five days before any surgery but if the patient has renal damage, it will take more than five days for complete clearance of the drug, i.e., almost 11 half-lives for 99.93% of the drug to be eliminated from the body [18]. But other factors play an important role in causing euglycemic DKA in addition to the drug itself. Alhemeiri et al. described a case where euglycemic DKA occurred even after the patient self-stopped the drug within 48 hours of his presentation because the patient had started following a ketogenic diet 72 hours before his presentation [19]. This shows that a ketogenic diet, a high fat, adequate protein, and low carbohydrate (< 50 grams) diet, is an additional factor in causing euglycemic DKA, as ketogenic diets result in high glucagon and low insulin levels along with increased levels of counterregulatory hormones like epinephrine and cortisol, which results in increased free fatty acid levels, which, in turn, increases ketone body production. Thus healthcare workers should counsel the patients regarding their diet before starting them on SGLT2 inhibitors. Pujara et al. have shown in their case report that glucosuria and ketonemia persisted nine to 10 days after stopping dapagliflozin, hence they recommend stopping the drug one week before any elective procedure [20], which indicates that the effect of the drug stays longer than expected based on its half-lives so we need more insight into the pharmacodynamics of the drug. Iqbal et al. described a case of euglycemic DKA being developed even after stopping dapagliflozin before two weeks of presentation even with normal renal function tests [21]. This shows that we need to take into account additional factors like genetic variation, drug-drug interaction, and other patient characteristics that can result in euglycemic DKA when deciding the time interval between drug stoppage and any elective procedure. We believe it will be helpful if we choose patients based on inclusion criteria for starting them on SGLT2 inhibitors, as mentioned in Table 2, to reduce the risk of euglycemic DKA.

**Table 2:** Inclusion criteria for patients SGLT2 - sodium-glucose cotransporter-2; eGFR - estimated glomerular filtration rate

INCLUSION CRITERIA FOR PATIENTS
Diagnosed with type 2 DM
Negative glutamic acid decarboxylase-antibody and islet cell antigen-2 antibodies
Age> 18 years
Follow diabetes appropriate diet regimen
No or moderate alcohol use
Non-pregnant
Counseled for regular blood glucose and serum ketones monitoring
No current illness, surgery, or dehydration
No gastrointestinal infection
No fasting or eating disorders
No renal failure/eGFR >60 ml/min/1.73 m2
Counseled regarding other medications that should not be taken with SGLT2 inhibitors

**Figure 2:**
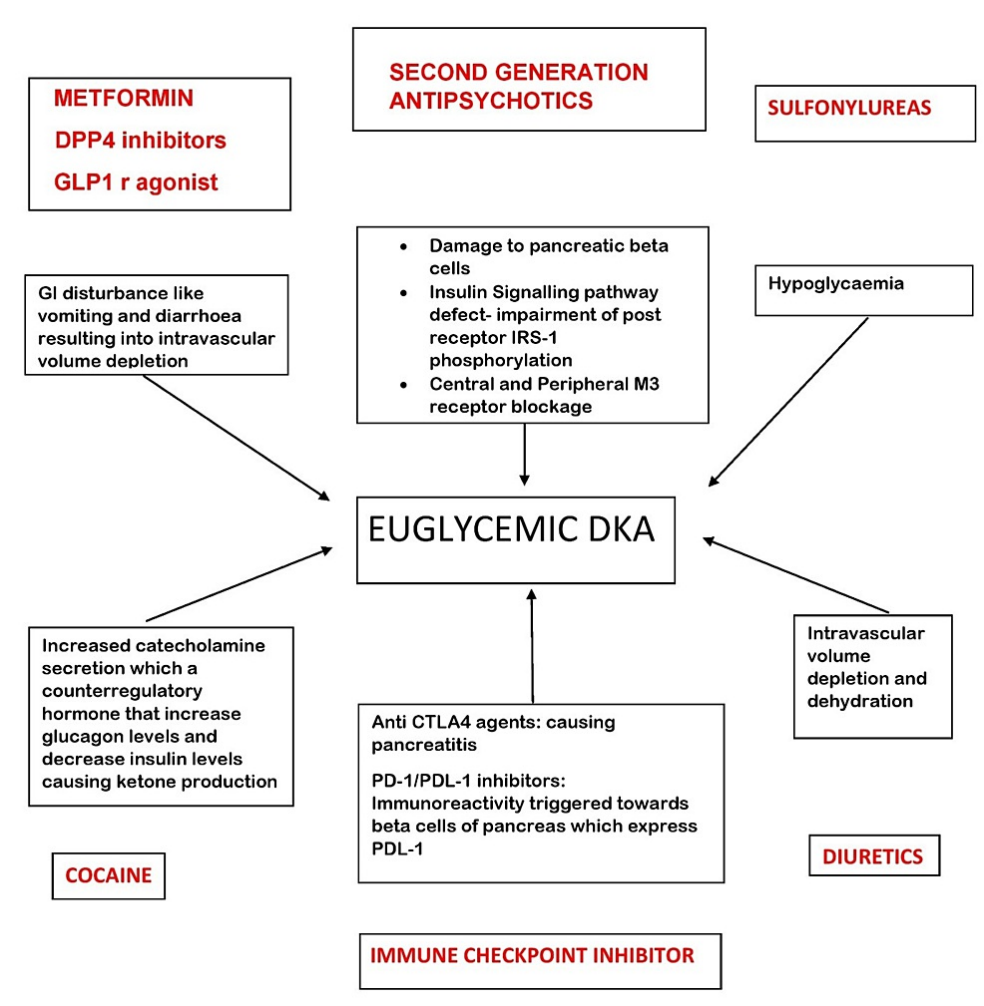
How different drugs with different mechanisms can result in euglycemic DKA when taken with SGLT2 inhibitors DPP4 - dipeptidyl peptidase 4; GLP1 r - glucagon-like peptide 1 receptor; IRS - insulin receptor substrate; CTLA4 - cytotoxic T lymphocyte-associated protein 4; PD - programmed death; PDL - programmed death ligand; SGLT2 - sodium-glucose cotransporter-2; DKA - diabetic ketoacidosis

When it comes to the drug withholding period after the patient has experienced euglycemic DKA, most agree with stopping the drug temporarily till the patient recovers but we do not have concrete guidelines for the withholding period as well. Parkin et al. mentioned that for patients who are switching their type of insulin therapy (e.g., injections to insulin pump therapy) or changing from manual mode to auto mode on an automated insulin delivery system, it is prudent that they hold their SGLT2 inhibitor until their insulin doses are adjusted, blood glucose is controlled, and ketone levels are normal [16]. The European Medicines Agency suggests stopping the treatment with SGLT2 inhibitors immediately if DKA is suspected and not starting the drug until a clear precipitating factor has been identified and resolved. It also advises doctors to not use SGLT2 inhibitors for type 1DM [22]. Lau et al. recommended postoperative SGLT2 inhibitors administration be withheld until adequate hydration and normal diet resumes and to consider bridging therapy with insulin/dextrose to prevent the development of euglycemic DKA [17]. It is only logical to stop the drug permanently if the patient experiences recurrent euglycemic DKA, but acknowledging comorbid cardiac conditions that patients may have and that the benefits of SGLT2 inhibitors outweigh their risk, continuing such patients on SGLT2 inhibitors is still an area that will need thorough research. Different drug interactions can also result in euglycemic DKA as explained in Figure 2.

Also, the literature suggests, it is important to review concomitant therapies to reduce the risk of euglycemic DKA via interaction with SGLT2 inhibitors [23-25]. Hence, patient counseling regarding this issue should be done by their primary care providers.

It is recommended that a multidisciplinary approach will be beneficial when dealing with SGLT2 inhibitor-induced euglycemic DKA and deciding its withholding period pre and post a euglycemic DKA episode. We encourage this decision to be made collectively by a team of endocrinologists, cardiologists, nephrologists, primary care physicians, and critical care physicians. Moreover, it is also important to counsel patients regarding appropriate dietary habits while taking the drug, i.e., not following low carbohydrate diets without consulting their primary care physicians. They should also be made aware of “sick day rules” where the patient is advised to not take the medication during acute illness [26]

Thorough research needs to be done in this area, which will help us develop a guideline for patients taking SGLT2 inhibitors.

## Conclusions

The purpose of this literature review is to provide data regarding existing guidelines for SGLT2 inhibitor-induced euglycemic DKA when it comes to stopping and restarting the drug. These guidelines vary among different institutions and different specialties based on the existing literature that we found; hence, we need more concrete research in this area to solidify the guidelines and solve the dilemma of whether to continue the drug after the euglycemic DKA episode. This literature review tries to inform us that we need to take into account patient inclusion criteria, concomitant therapies, thorough patient counseling regarding precipitating factors, and easy access to health care, as delayed treatment in case of severe acidosis can be fatal when deciding the guidelines for restarting the drug after a euglycemic DKA episode.
